# The Comorbidity of Gambling Disorder among Macao Adult Residents and the Moderating Role of Resilience and Life Purpose

**DOI:** 10.3390/ijerph15122774

**Published:** 2018-12-07

**Authors:** Juliet Honglei Chen, Kwok Kit Tong, Anise M. S. Wu, Joseph T. F. Lau, Meng Xuan Zhang

**Affiliations:** 1 Department of Psychology, Faculty of Social Sciences, University of Macau, Avenida da Universidade, Taipa, Macao, China; juliethchen@outlook.com (J.H.C.); kktong@um.edu.mo (K.K.T.); yb77304@connect.um.edu.mo (M.X.Z.); 2 The Jockey Club School of Public Health and Primary Care, The Chinese University of Hong Kong, Hong Kong, China; jlau@cuhk.edu.hk

**Keywords:** gambling disorder, Internet gaming disorder, depression, anxiety, comorbidity, prevalence, psychological resilience, purpose in life, Chinese, adults

## Abstract

Macao, China’s only city with legalized casinos, has maintained a high prevalence of gambling participation and gambling disorder (GD) over the past decade. The mental health risks associated with such high levels have been overlooked. In order to estimate the comorbid prevalence of GD with depression, anxiety, and Internet gaming disorder (IGD) and to explore the potential buffering effect of psychological resilience and purpose in life, this study obtained a representative adult Chinese sample (*N* = 1000, 44% male, aged 18–97 years) from a telephone survey conducted between October and November of 2016. As hypothesized, the highest psychiatric comorbid prevalence was observed in the GD subgroup (*n* = 19, 21.1% probable IGD, 26.3% probable depression, and 37.0% probable anxiety). All these mental health problems could increase one’s proclivity to GD, and vice versa. Psychological resilience was found to buffer the association between anxiety symptoms and probable GD (χ^2^(1) = 4.30, *p* = 0.04/GD symptoms, *F*_change_ (1,162) = 6.29, *p* = 0.01), whereas purpose in life did not display any hypothesized moderating effect. These results indicate the usefulness of mental health screening for GD, taking into consideration its associated risks, and of fostering psychological resilience in prevention and treatment programs.

## 1. Introduction

Macao, with its reputation as the Las Vegas in the East and with its over 170 years of gambling industry history, is the only city in the greater China territory with legalized casino gambling since its sovereignty was returned to China, from Portugal, in 1999 [1,2]. Over the past 10 years, an estimated 49.5% to 55.9% (*N* = 1963 to 2158) of its residents (≥ 15 years old) have participated in at least one gambling activity, of which 0.7% to 1.3% were identified as probable DSM-IV pathological gamblers in earlier years and 2.5% were identified as probable DSM-5 disordered gamblers in 2016 [3,4,5,6]. Similar prevalence has been reported in other local studies, such as 5.6% probable pathological gambling in 2010 (*N* = 2011) [7] and 2.1% probable disordered gambling in 2014 (*N* = 1018) [8]. These estimated percentages are considerably higher than those in other regions that have legalized casino gambling, such as a low 0.1% probable disordered gambling in Singapore (*N* = 3000) [9]. The current study sought to make use of Macao’s solid residential gambling participation and to respond to an elevated call from the community to examine the risks associated with the high rates of gambling disorder (GD), as well as to look for potential buffers.

Other than demographic risks, such as male, younger, and with lower education levels [7,8,10], previous research has identified depression and anxiety as the two most frequently reported forms of mental distress associated with being a gambler rather than a non-gambler [11,12,13]. In a recent systematic review of 11 general populations (*N* = 2417 to 43,093), an average comorbidity of 37.9% for mood disorders (including major depression and bipolar disorder/manic episodes) and 37.4% for anxiety were found to have gambling problems, including both problem gambling and pathological gambling [14]. Depression and anxiety in disordered gamblers were found to increase the likelihood of health problems such as sleep disturbances [15], which are associated with risk behaviors including substance use and lethal suicide attempts [16,17]. In another retrospective study (*N* = 9282), prior DSM-IV mood and anxiety disorders were found to predict the onset and persistence of pathological gambling; 74.3% of the pathological gamblers with another lifetime mental disorder had an earlier onset of at least one such disorder that preceded pathological gambling [10]. However, the local comorbidity of GD and emotional distress remains unstudied in Macao, hence, the potential risk of GD to the community’s mental health, and vice versa, is highly likely to be underestimated. The first objective of the present study was to estimate the comorbidity between GD and its two most common forms of emotional distress (i.e., depression and anxiety) to provide more accurate statistics for clinical practice and community health promotion.

When exploring the relationship between GD and other types of behavioral addictions, similarities between GD and Internet gaming disorder (IGD) were speculated and examined [18], with the latter even being called a “nonfinancial form of gambling” (p.54) [19]. Although a positive relationship between the two has been consistent [20,21], the comorbidity of the two appears to be an understudied area of inquiry. The only relevant study examined the comorbidity of video gaming dependence and DSM-IV pathological gambling, which identified 15% of 193 treatment-seeking pathological gamblers as having video dependence [22]. On the other hand, Dowling and Brown, using an Australian college sample (*N* = 173), reported that there was no overlap between problem gambling and Internet dependence [23]. It is not clear whether the inconsistency in these findings is due to the different samples or to the different diagnostic tools used. To fill the gaps in the existing literature regarding DSM-5-based comorbidity between GD and IGD, our second objective was to examine the hypothesized co-occurrence between the two disorders in order to provide more empirical data for further comparisons by behavioral addiction scientists and practitioners.

Our third objective was to explore the potential buffering effects of two personal strength constructs (i.e., psychological resilience and purpose in life) on the relationship between GD and its comorbidities to improve public health promotion and policy design efforts. Although no existing empirical studies have directly evaluated the moderating effects of these two, indirect evidence indicates that resilience may play a protective role against problem gambling in the general population and among adolescents [24,25]. Resilience has also been found to have a negative association with both emotional distress and addictive behaviors, such as substance use [26]. In a grounded-theory study, Holdsworth reported that unlike recreational gamblers, problem gamblers appeared to lack the resilience necessary to deal with life events and psychological comorbidities, which hints at the possible protective role of resilience against GD and its comorbidities [27]. Purpose in life could potentially perform a similar buffering role, which was found indirectly from its negative correlation with addictive behaviors, including problem gaming [28], Internet addiction [29], and smoking [30]. To the best of our knowledge, no study has tested or documented the moderating effect of either psychological resilience or purpose in life on the association between disordered gambling and other mental problems, such as emotional distress and IGD.

Altogether, the present study hypothesized (a) the co-occurrence of GD with depression, anxiety, and IGD in the general population of Macao, and (b) the buffering effect of psychological resilience and purpose in life on the relationship between GD and its comorbidities in Macao adult residents. Our findings would (a) set the groundwork for subsequent DSM-5-based comorbidity studies of GD, (b) highlight the potentially increased risks of GD to draw the public’s attention to the need for early prevention and treatment, and (c) identify protective buffers that may alleviate the impact of comorbid psychiatric symptoms (if any) of GD for practitioners and policy advocates.

## 2. Materials and Methods

### 2.1. Respondents and Procedures

Between October and November of 2016, we conducted a random sampling telephone survey among Chinese Macao residents based upon the 2015 Macao residential phonebook. Both male and female Chinese Macao residents who were 18 years old or above were included. We adopted a two-step stratified random sampling method of phone poll studies. We randomly selected eligible residential units in the first step, following which we selected a household member based on the last birthday rule. In the end, 1000 respondents voluntarily participated in and completed the approximate 12-minute phone survey without any monetary incentives, which incurred an overall 61.8% cooperation rate by the standard of American Association for Public Opinion Research [31].

The overall sample had more females (56%; 95% CI [52.9%, 59.1]), than males (44%; 95% CI [40.9%, 47.1]), and a mean age of 40.0 years (*SD* = 15.3; ranging from 18 to 97), which was similar in sex and age distribution to the population parameters of 2016 as reported by the census of Macao [32]. The sample consisted of 68.0% secondary or tertiary graduates, 66.9% full-time or part-time workers, and 11.9% casino employees. A total of 183 respondents who had engaged in gambling in the past 12 months were classified as “recent gamblers”. 

This study was part of a research project that had obtained approval from the ethics committee of the affiliated university of the corresponding author (MYRG2015-00213-FSS). Using the same dataset, the findings about prevalence and correlates of problematic adult gaming in Macao was reported in another published paper [33].

### 2.2. Measures Section

For probable GD, consistent with previous studies [7,8,34], we used the nine DSM-5 diagnostic criteria for GD to assess illness symptoms and identify probable disordered gamblers [35]. Only respondents with past-year gambling experience (*n* = 183) answered whether each of the symptoms (e.g., preoccupation with gambling) described their own condition within the last year (0 = *no*, 1 = *yes*). Internal consistency (KR-20) of these items was 0.81 for the current sample. A total sum of the scale score corresponded to the GD symptom variables (ranging from 0 to 9). A cutoff of 3/4 was used, in accordance with guidelines of the DSM-5 [35], to create the probable GD variable (1 = *no*, 2 = *yes*). 

Depression and anxiety symptoms were independently measured with two 7-item subscales from the Chinese version of the 21-item Depression Anxiety Stress Scales (DASS-21) [36]. It is with a 4-point Likert scale, in which 0 = *did not apply to me at all*, and 3 = *applied to me very much or most of the time*. A total depression score (i.e., depression symptoms) and a total anxiety score (i.e., anxiety symptoms), which both ranged from 0 to 42, were computed. Higher total depression scores indicated more depressive symptoms (Cronbach’s α = 0.82). Consistent with previous studies [37,38], respondents with a score of ≥14 (moderate and above) were classified as probable depressed cases. Similarly, higher total anxiety scores represented higher levels of anxiety (Cronbach’s α = 0.79), with a cut-off of ≥10 (moderate and above) to classify probable cases with anxiety [37,38].

Probable IGD symptoms were measured with the nine DSM-5 diagnostic criteria for this disorder [35], which is in accordance with previous research [39]. Only 473 self-reported past-year gamers were asked to respond to these items about their experience of problematic gaming (0 = *no*, 1 = *yes*) in the past 12 months (KR-20 = 0.69). The total sum scale score ranged from 0 to 9, which corresponded to the variables of IGD symptoms in the subsequent analyses. Another variable, probable IGD, was computed, with a cutoff of 4/5, to differentiate probable IGD respondents from non-IGD respondents [40].

Psychological resilience was measured via the 10-item Chinese version of the Connor–Davidson Resilience Scale [41,42]. It is a 5-point Likert scale, in which 0 = *not true at all* and 4 = *true nearly all the time*. Higher scale scores indicated higher resilience levels, with the Cronbach’s α = 0.90 in this study.

Perceived purpose in life was assessed with the 6-item version of Crumbaugh and Maholick’s Purpose in Life Scale with a 5-point Likert scale (1 = *strongly disagree* to 5 = *strongly agree*) [43,44]. Higher scores reflected higher levels of positivity in perceived life purpose, with the Cronbach’s α = 0.82 in this study.

The background variables reported by respondents were their age, sex, education level, employment status, and casino employment status. Regarding gambling expenses, recent gamblers were asked to decide how much they had spent during each gambling incident. 

### 2.3. Data Analysis

All statistical analyses were conducted with SPSS 25.0 (IBM, Armonk, NY, USA) [45]. We first examined the descriptive statistics and inter-correlations of all the key variables and categorized the overall sample into two groups of sub-samples, namely, non-gamblers versus recent gamblers and non-GD gamblers versus probable GD gamblers. Multiple between-group comparisons were conducted to explore potentially significant characteristic differences with χ^2^ tests and Mann–Whitney tests for categorically scaled (e.g., working status), ordinally scaled (e.g., educational attainment), or skewed variables (e.g., age), and *t*-tests were conducted for continuous variables. Based upon the sub-group comparison results, we calculated the Prevalence Ratio (*PR* = $\frac{P\;\left(with\;diease\right)}{P\;\left(with\;exposure\right)}$) to indicate the comorbid risk of GD with depression, anxiety, and IGD.

Finally, we explored the potential moderating effects of psychological resilience and purpose in life on the relationship between GD (i.e., probable GD and GD symptoms) and its three co-occurring mental health problems (i.e., depression, anxiety, and IGD), respectively, with six hierarchical ordinary least squares linear regressions for GD symptoms and six hierarchical maximum likelihood binary logistic regressions for probable GD. In each hierarchical model, two well-established risky demographic factors, age and gender [46], were controlled for as covariates in the first step. In the second step, the focal predictive variables (i.e., one comorbid symptom variable and one personal strength variable) were entered, and in the last step, the interaction term of the focal predictive variables was entered. All of the focal predictive variables and their product terms in these moderation models were mean-centered and bootstrapping (*n* = 5000) was performed to reduce bias [47].

## 3. Results Section

### 3.1. Profiles of this Sample

The overall sample (*N* = 1000) was categorized into non-gamblers (*n* = 817) versus recent gamblers (*n* = 187; with 168 non-GD gamblers [89.8%] and 19 probable GD gamblers [10.2%]). Table 1 shows that males (*OR* = 2.96) and lower educational levels (*OR* = 1.47 [Junior secondary and lower versus Senior secondary and higher]) were more likely to engage in gambling in the past year. This pattern was also observed among probable GD gamblers compared to non-GD gamblers (*OR* = 3.04 for males; *OR* = 3.43 for less educated). 

Probable GD gamblers, relative to non-GD gamblers, reported spending significantly more money on gambling (*p* < 0.001) and showed a significantly higher tendency to suffer from IGD (*OR* = 12.53) and from depression (*OR* = 4.15). A similar online gaming engagement pattern was found among past-year gamblers, in which they were more likely to play online games than non-gamblers, χ^2^(1) = 17.14, *p* < 0.001, *OR* = 1.98. However, it is worth noting that working in casinos did not significantly affect whether the respondents gambled in the past year (*p* = 0.97) or show probable GD (*p* = 0.22). Non-gamblers and recent gamblers, moreover, did not have significantly different levels of probable IGD/anxiety/depression (*p* > 0.05).

### 3.2. Comorbidity Analysis

In the overall sample, the prevalence of probable GD, probable IGD, probable depression, and probable anxiety was 1.9% (95% CI [1.05, 2.74]), 2.0% (95% CI [1.13, 2.87]), 11.3% (95% CI [9.34, 13.26]), and 20.1% (95% CI [17.62, 22.58]), respectively. When looking into the recent gambler subgroup (*n* = 183), the prevalence of all factors increased significantly, to 10.4% (95% CI [5.96, 14.80]) probable GD, 3.8% (95% CI [1.05, 6.60]) probable IGD, 9.8% (95% CI [5.52, 14.15]) probable depression, and 22.4% (95% CI [16.36, 28.45]) probable anxiety. The prevalence of these four mental health disorders peaked within the probable GD subgroup (*n* = 19), in which probable IGD was 21.1% (95% CI [2.72, 39.38]), probable depression was 26.3% (95% CI [6.52, 46.12]), and probable anxiety was 37.0% (95% CI [15.15, 58.53]). The Prevalence Ratio (PR) was further computed to depict the comorbid relationship between GD and the other three mental problems. Among the probable GD population, the PR of probable IGD, probable depression, and probable anxiety was 5.94, 3.27, and 2.02, respectively. Conversely, the PR of probable GD was 9.23, 3.32, and 1.78 in probable IGD, probable depression, and probable anxiety subgroups, respectively. 

### 3.3. Exploring Buffering Effects of Psychological Resilience and Purpose in Life

**Figure 1:**
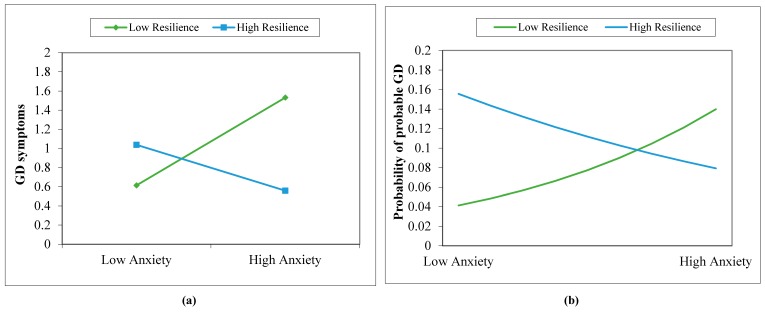
Moderating effects of psychological resilience on the relationship between anxiety and (**a**) GD symptoms and (**b**) probable GD. The focal variables of anxiety symptoms and resilience were mean-centered and adopted +1SD as the high point and –1SD as the low point, respectively.

Table 2 presents the results of the analyses regarding the potential moderating effects of psychological resilience and purpose in life on the associations between GD (i.e., GD symptoms and probable GD) and each of the three comorbid disorder symptoms in the study. As shown, one set of six independent moderation models was built to assess the six product terms (i.e., depression symptoms × purpose, depression symptoms × resilience, anxiety symptoms × purpose, anxiety symptoms × resilience, IGD symptoms × purpose, and IGD symptoms × resilience) in moderating between symptoms of depression/anxiety/IGD and GD symptoms. Similarly, another set of six models was examined for probable GD. The results showed no statistical significance for most of the interaction terms, with the exception of anxiety symptoms × resilience, which displayed a consistent moderating effect on the relationship between anxiety and GD symptoms (*R*^2^_Change_ = 0.035, *F*_change_ (1,162) = 6.29, *p* = 0.01) and between anxiety and probable GD (χ^2^(1) = 4.30, *p* = 0.04), respectively. We further probed these moderating effects with a simple slopes analysis [48,49], and the results are presented in Figure 1.

## 4. Discussion Section

In this study, we collected a representative adult sample of the Macao community and observed 19 probable GD gamblers, representing 10.4% (95% CI [5.96, 14.80]) in our past-year gambler sub-sample (*n* = 187), which is higher than the prevalence reported in other larger local gambler samples, such as 7.4% out of 282 past-year gamblers [8] and 4.9% of 1030 past-year gamblers [6]. Such a high prevalence of probable GD, reported across studies and time both in the general and gambler samples, shows that gambling disorder is persistent and alarming in Macao, warranting further research and clinical attention. 

Our findings concerning the demographic profiles among recent gamblers and probable GD gamblers, such as males with lower educational attainment, are consistent with former local studies [7,8]. The amount of gambling expense was consistently found to be a satisfactory indicator of probable GD gamblers when compared to non-GD gamblers, in this study and previous studies [8]. However, casino employment no longer appeared to be a risk factor for probable GD, which might be attributed to the implementation of responsible gambling training programs for casino workers and local residents in recent years [50,51]. In addition, non-disordered gambling did not appear to increase the risk for IGD, depression, or anxiety, as evidenced by the lack of significant results when comparing non-gamblers with past-year gamblers. This finding lends some empirical support to the possibility that responsible gambling policy may reduce the potential harm of gambling on the public’s mental health. However, further resources must be allocated to screening and treatment for those with GD or those at-risk.

When comparing the prevalence of probable IGD, probable depression, and probable anxiety in the three samples (i.e., the overall, the recent gambler, and the probable GD), the prevalence of all three forms of mental health problems peaked in the probable GD sample. To quantify the increased risk level, we calculated PR for further comparison. As a result, compared to the non-GD group, people with GD were 5.94 times, 3.27 times, and 2.02 times more likely to have probable IGD, probable depression, and probable anxiety, respectively. In contrast, the risk of having probable GD would be 9.23 times more likely among probable IGD cases when compared to non-IGD cases, 3.32 times more likely among probable depression cases when compared to non-probable depression cases, and 1.78 times more likely among probable anxiety cases when compared to non-probable anxiety cases. These data confirmed the co-occurrence of these mental problems and provided extra support to previous studies’ claims regarding positive correlations between GD and other mental health disorders [10]. They also confirmed that problem gambling could be initiated by depression and anxiety [52].

Although the mutual risks of having probable GD in emotionally distressed populations and having emotional distress in probable GD populations are comparable, probable IGD cases were found to be at a much higher risk of suffering from probable GD (*PR* = 9.23) than probable GD cases were of suffering from probable IGD (*PR* = 5.94). In addition to the likelihood of a high co-occurrence, due to similar mechanisms for the development of these two types of behavioral addictions, our findings may also reflect the notion that IGD may be a “gateway illness” for GD development. Further empirical evidence is needed to confirm this speculation. The findings may also be attributed to different determinants involved in the development of these two behavioral addictions, which may be related to gambling’s essential financial elements [18], as well as to gaming pursuits placing more focus on skill [53].

A statistically significant buffering effect was found only in relation to psychological resilience, which moderated the relationship between anxiety symptoms and probable GD (in both binary and continuous forms). Specifically, even after controlling for the effects of age and gender, the buffering effects of psychological resilience still explained 3.5% of the variance in the association between anxiety and GD symptoms, and decreased the risk of having probable GD from anxiety by 0.89 times (Bias-Corrected and accelerated [BCa] bootstrap 95% CI [0.73, 0.98]). From the two simple probing analyses in the figures, we inferred that those with higher levels of resilience had a lower probability of suffering from GD symptoms and being a probable GD gambler, even when anxiety levels were high. Given that similar buffering patterns were found in both GD symptoms and probable GD, we inferred that the protective effect of resilience can work for both general gamblers and probable GD gamblers. This finding also extended the protective function of psychological resilience to probable GD gamblers from previously discovered recreational gamblers [27]. However, a similar buffering effect of psychological resilience was not found in the relationship between depression and GD symptoms/probable GD. One plausible reason for lacking a buffering effect on depression is that depression, compared to anxiety, might be non-linearly related to GD symptoms/probable GD. For example, in a two-year longitudinal study of 6067 casino employees, respondents who gambled and had severe and disabling depression (i.e., presenting dysfunctions at work or in personal life for at least two weeks over the past six months) reported a reduced level of gambling problems about one year later [54]. It is possible that gamblers with serve depression may be less responsive to external influences (including stressors), probably because of their low energy levels, and thus psychological resilience may also lose its buffering role. Further research is needed to understand the mechanisms underlying the GD-depression relationship and to identify potential buffers in such relationship.

The buffering effects of purpose in life on the relationships between GD symptoms/probable GD and any of its comorbidities were not observed. A plausible reason was that its buffering effect was cancelled out by other uncontrolled confounding variables. One possible confounding variable was the extent to which people search for meaning in life. A previous study showed that the predictive value of meaning/purpose on anxiety and perceived health disappeared among the respondents reporting a low level of search for meaning [55]. Thus, we would call for more replication studies examining the potential protective roles of not only purpose in life but also psychological resilience, their relationships with GD, and their comorbidities after considering other possible confounding variables.

Although we found a co-occurrence of GD with several mental health problems and discovered some promising future directions, our study presented several limitations that are worth noting. First, although we found a co-occurrence of probable GD, probable IGD, probable depression, and probable anxiety, and calculated a PR for each condition, causal inferences cannot be made because we did not have control over the precedence of any of the events or conditions in this correlational study. Longitudinal studies are strongly recommended to further explore which conditions appear to emerge first with regard to GD and its psychiatric comorbidities. Second, collecting data in the form of a telephone survey is a double-edged sword. Although this methodology assured a representative sample, only non-clinical diagnostic tools were used in this survey and they were only able to identify probable cases of the concerned mental disorders. The fact that the survey was administrated over the telephone necessarily set restrictions on length. Hence, we had to choose shorter versions of the scales and sacrificed other valuable constructs, such as search for meaning. Third, although we tried to minimize the self-report bias by assuring respondents of the anonymous nature of the survey at the beginning of each phone interview, there may still have been some lingering effects of self-report bias in our findings, which may also occur in other survey studies using self-report methods.

## 5. Conclusions

As the first DSM-5 criteria comorbidity study of probable GD with probable depression/anxiety/IGD in a representative Chinese adult sample, this study underscores the increased susceptibility to developing other mental disorders among probable GD gamblers and thus provides practical insights for mental health screening and prevention for practitioners and policy makers. Specifically, assessment of GD, IGD, depression, and anxiety are recommended to be conducted together in general population screenings and in psychiatric diagnostic interviews of GD gamblers. The designers of GD intervention programs should be aware of any co-occurred psychological distress and problem gaming patterns and adopt a more integrated approach that addresses not only GD but also its co-morbidity, simultaneously. The buffering effect of psychological resilience between anxiety and GD symptoms/probable GD sheds light on the potential usefulness of fostering psychological resilience as part of prevention and treatment programs. In addition to testing the replicability of the current findings, future studies should also investigate the underlying mechanism between GD and other mental disorders, and the effectiveness of interventions that incorporate the factors examined in this study.

## Figures and Tables

**Table 1:** Comparing Stratified Profiles of Overall Sample Subgroups and Gambler Subgroups.

	Overall Sample Subgroups		Gambler Subgroups	
	Non-Gambler (*n* = 817)	Recent Gambler (*n* = 183)	Non-GD (*n* = 168)	Probable GD (*n* = 19)
Age	U = 56297.00, z = −1.63, *p* = 0.10		U = 1075.50, z = −1.10, *p* = 0.27	
[M (SD)]	39.60 (15.39)	41.51 (15.11)	41.09 (14.87)	45.29 (17.13)
Sex	χ^2^(1) = 42.31, *p* < 0.001		χ^2^(1) = 3.26, *p* = 0.07	
[Male]	39.2%	65.6%	63.4%	84.2%
Educational attainment	U = 64558.00, z = −1.99, *p* = 0.05		U = 884.50, z = −2.56, *p* = 0.01	
Primary or below	13.1%	20.0%	17.7%	41.3%
Junior secondary	15.1%	16.7%	16.0%	23.5%
Senior secondary	28.3%	24.4%	25.2%	17.6%
Tertiary	43.5%	38.9%	41.1%	17.6%
Working status	χ^2^(3) = 3.49, *p* = 0.32		χ^2^(3) = 1.39, *p* = 0.71	
Full-time	62.0%	65.2%	64.4%	72.2%
Part-time	5.7%	7.7%	7.4%	11.1%
Student	9.6%	6.1%	6.1%	5.6%
Other	22.7%	21.0%	22.1%	11.1%
Casino Employee	χ^2^(1) = 0.002, *p* = 0.97		χ^2^(1) = 1.54, *p* = 0.22	
[Yes%]	11.6%	13.1%	13.7%	5.3%
Gambling expense ^a^	−		U = 671.50, z = −3.59, *p* < 0.001	
[Mdn]	−	$12.37–61.76	$12.37–61.76	$123.76–247.40
Resilience	*t* (996) = −0.10, *p* = 0.92		*t* (181) = 0.12, *p* = 0.91	
[M (SD)]	2.59 (0.67)	2.59 (0.68)	2.59 (0.68)	2.57 (0.70)
Purpose in life	*t* (998) = −0.90, *p* = 0.37		*t* (181) = 0.41, *p* = 0.69	
[M (SD)]	3.36 (0.64)	3.32 (0.61)	3.32 (0.60)	3.26 (0.65)
**Mental health problems**				
Depression	χ^2^(1) = 0.48, *p* = 0.49		χ^2^(1) = 9.49, *p* = 0.01	
DASS < 14 [n (%)]	722 (88.4%)	165 (90.2%)	151 (92.1%)	14 (73.7%)
DASS ≥ 14 [n (%)]	95 (11.6%)	18 (9.8%)	13 (7.9%)	5 (26.3%)
Anxiety	χ^2^(1) = 0.27, *p* = 0.61		χ^2^(1) = 2.54, *p* = 0.11	
DASS < 10 [n (%)]	648 (79.3%)	142 (77.6%)	130 (79.3%)	12 (63.2%)
DASS ≥ 10 [n (%)]	169 (20.7%)	41 (22.4%)	34 (20.7%)	7 (36.8%)
IGD ^b^	χ^2^(1) = 1.47, *p* = 0.23		χ^2^(1) = 13.44, *p* < 0.001	
DSM-5 < 5 [n (%)]	344 (96.4%)	104 (93.7%)	94 (96.9%)	10 (71.4%)
DSM-5 ≥ 5 [n (%)]	13 (3.6%)	7 (6.3%)	3 (3.1%)	4 (28.6%)

*Note*. GD—Gambling Disorder, IGD—Internet Gaming Disorder. ^a^ The gambling expense has been converted from local currency to US dollars. ^b^ The total IGD sample (n = 468) includes only past-year online gamers.

**Table 2:** Exploring Potential Moderating Effects on GD Symptoms and Probable GD Models.

Models	GD Symptoms		Probable GD	
Moderating Effect Terms	*b* [95% CI] ^a^	Interaction Effect	*OR* [95% CI] ^a^	Interaction Effect
1. Depression symptoms × purpose	0.04 [−0.04, 0.07]	*R*^2^_Change_ = 0.02, *F* (1,162) = 3.65, *p* = 0.06	1.08 [0.89, 1.17]	χ^2^(1) = 3.30, *p* = 0.07
2. Depression symptoms × resilience	−0.01 [−0.11, 0.03]	*R*^2^_Change_ = 0.001, *F* (1,162) = 0.18, *p* = 0.68	0.98 [0.86, 1.06]	χ^2^(1) = 0.13, *p* = 0.72
3. Anxiety symptoms × purpose	0.03 [−0.07, 0.07]	*R*^2^_Change_ = 0.01, *F* (1,162) = 1.92, *p* = 0.17	1.06 [0.84, 1.17]	χ^2^(1) = 1.86, *p* = 0.17
4. Anxiety symptoms × resilience	−0.08 [−0.16, −0.004]	*R*^2^_Change_ = 0.035, *F* (1,162) = 6.29, *p* = 0.01	0.89 [0.79, 0.94]	χ^2^(1) = 4.30, *p* = 0.04
5. IGD symptoms × purpose	−0.01 [−0.72, 0.27]	*R*^2^_Change_ < 0.001, *F* (1,98) = 0.01, *p* = 0.92	1.29 [0.26, 2.27]	χ^2^(1) = 1.06, *p* = 0.30
6. IGD symptoms × resilience	−0.10 [−0.73, 0.16]	*R*^2^_Change_ = 0.004, *F* (1,98) = 0.44, *p* = 0.51	1.17 [0.38, 2.44]	χ^2^(1) = 0.38, *p* = 0.54

*Note*. GD—Gambling disorder, IGD—Internet Gaming Disorder. In each model, gender and age were controlled for as covariates, either GD symptoms or probable GD were tested as the dependent variable. All the focal predictive variables were mean-centered continuous variables. ^a^ Bootstrapping results were based on 5000 bootstrap samples. Biased-Corrected and accelerated (BCa) bootstrap 95% confidence intervals (CI) were reported.

## References

[1] Chan P.K., Chan P.C. (2001). Gaming Industry and its Opportunities for Development.

[2] Wu AM S., Lau JT F. (2015). Gambling in China: Socio-historical evolution and current challenges. Addiction.

[3] Institute for the Study of Commercial Gaming (ISCG) (2008). Report on a Study of Macao People’s Participation in Gambling Activities 2007.

[4] Institute for the Study of Commercial Gaming (ISCG) (2010). Report on a Study of Macao People’s Participation in Gambling Activities 2010.

[5] Institute for the Study of Commercial Gaming (ISCG) (2014). Report on a Study of Macao People’s Participation in Gambling Activities 2013.

[6] Institute for the Study of Commercial Gaming (ISCG) (2016). Report on a Study of Macao People’s Participation in Gambling Activities 2016.

[7] Fong D.K.-C., Ozorio B. (2005). Gambling participation and prevalence estimates of pathological gambling in a far-east gambling city: Macao. UNLV Gaming Res. Rev. J..

[8] Wu A.M.S., Lai M.H.C., Tong K.K. (2014). Gambling disorder: Estimated prevalence rates and risk factors in Macao. Psychol. Addict. Behav..

[9] National Council on Problem Gambling (NCPG) (2018). Report of Survey on Participation in Gambling Activities among Singapore Residents 2017.

[10] Kessler R.C., Hwang I., LaBrie R., Petukhova M., Sampson N.A., Winters K.C., Shaffer H.J. (2008). DSM-IV pathological gambling in the National Comorbidity Survey Replication. Psychol. Med..

[11] Boughton R., Falenchuk O. (2007). Vulnerability and comorbidity factors of female problem gambling. J. Gambl. Stud..

[12] Cunningham-Williams R.M., Cottler L.B., Compton WM I.I.I., Spitznagel E.L. (1998). Taking chances: Problem gamblers and mental health disorders-results from the St. Louis Epidemiologic Catchment Area Study. Am. J. Public Health.

[13] El-Guebaly N., Patten S.B., Currie S., Williams J.V.A., Beck C.A., Maxwell C.J., Wang J.L. (2006). Epidemiological associations between gambling behavior, substance use & mood and anxiety disorders. J. Gambl. Stud..

[14] Lorains F.K., Cowlishaw S., Thomas S.A. (2011). Prevalence of comorbid disorders in problem and pathological gambling: Systematic review and meta-analysis of population surveys. Addiction.

[15] Parhami I., Siani A., Rosenthal R.J., Lin S., Collard M., Fong T.W. (2012). Sleep and gambling severity in a community sample of gamblers. J. Addict. Dis..

[16] Pompili M., Innamorati M., Forte A., Longo L., Mazzetta C., Erbuto D., Ricci F., Palermo M., Stefani H., Seretti M.E. (2013). Insomnia as a predictor of high-lethality suicide attempts. Int. J. Clin. Pract..

[17] Taylor D.J., Lichstein K.L., Durrence H.H. (2003). Insomnia as a health risk factor. Behav. Sleep Med..

[18] King D.L., Delfabbro P.H. (2018). Internet Gaming Disorder: Theory, Assessment, Treatment, and Prevention.

[19] Griffiths M.D. (1991). Amusement machine playing in childhood and adolescence: A comparative analysis of video games and fruit machines. J. Adolesc..

[20] Parker J.D.A., Taylor R.N., Eastabrook J.M., Schell S.L., Wood L.M. (2008). Problem gambling in adolescence: Relationships with internet misuse, gaming abuse and emotional intelligence. Pers. Indiv. Differ..

[21] Walther B., Morgenstern M., Hanewinkel R. (2012). Co-occurrence of addictive behaviours: Personality factors related to substance use, gambling and computer gaming. Eur. Addict. Res..

[22] Jimenez-Murcia S., Fernandez-Aranda F., Granero R., Choliz M., La Verde M., Aguglia E., Signorelli M.S., Sa G.M., Aymami N., Gomez-Pena M. (2014). Video Game Addiction in Gambling Disorder: Clinical, Psychopathological, and Personality Correlates. Biomed. Res. Int..

[23] Dowling N.A., Brown M. (2010). Commonalities in the psychological factors associated with problem gambling and Internet dependence. Cyberpsychol. Behav. Soc. Netw..

[24] Oei T.P., Goh Z. (2015). Interactions Between Risk and Protective Factors on Problem Gambling in Asia. J. Gambl. Stud..

[25] Lussier I., Derevensky J.L., Gupta R., Bergevin T., Ellenbogen S. (2007). Youth gambling behaviors: An examination of the role of resilience. Psychol. Addict. Behav..

[26] Fumaz C.R., Ayestaran A., Perez-Alvarez N., Munoz-Moreno J.A., Molto J., Ferrer M.J., Clotet B. (2015). Resilience, ageing, and quality of life in long-term diagnosed HIV-infected patients. AIDS Care.

[27] Holdsworth L., Nuske E., Hing N.A. (2015). Grounded Theory of the Influence of Significant Life Events, Psychological co-Morbidities and Related Social Factors on Gambling Involvement. Int J. Ment. Health Addiction.

[28] Wu A.M.S., Lei L.L.M., Ku L. (2013). Psychological needs, purpose in life, and problem video game playing among Chinese young adults. Int. J. Psychol..

[29] Zhang Y., Mei S., Li L., Chai J., Li J., Du H. (2015). The relationship between impulsivity and internet addiction in Chinese college students: A moderated mediation analysis of meaning in life and self-esteem. PLoS ONE.

[30] Thege B.K., Bachner Y.G., Kushnir T., Kopp M.S. (2009). Relationship between meaning in life and smoking status: Results of a national representative survey. Addict. Behav..

[31] American Association for Public Opinion Research (AAPOR) (2016). Standard Definitions: Final Dispositions of Case Codes and Outcome Rates for Surveys.

[32] Macao Statistics and Census Services (2017). Detailed Results of 2016 Population By-Census. Macao: Macao Statistics and Census Services.

[33] Wu AM S., Chen J.H., Tong K.K., Yu S., Lau JT F. (2018). Prevalence and associated factors of Internet gaming disorder among community dwelling adults in Macao, China. J. Behav. Addict..

[34] Wu A.M.S., Tang C.S. (2012). Problem gambling of Chinese college students: Application of the theory of planned behavior. J. Gambl. Stud..

[35] American Psychiatric Association (2013). Diagnostic and Statistical Manual of Mental Disorders.

[36] Moussa M.T., Lovibond P.F., Laube R. (2001). Psychometric properties of a Chinese version of the short Depression Anxiety Stress Scales (DASS21). Report for New South Wales Transcultural Mental Health Centre.

[37] Carter F.A., Bell C.J., Ali A.N., McKenzie J., Wilkinson T.J. (2014). The impact of major earthquakes on the psychological functioning of medical students: A Christchurch, New Zealand study. Med. J..

[38] Kulsoom B., Afsar N.A. (2015). Stress, anxiety, and depression among medical students in a multiethnic setting. Neuropsych. Dis. Treat..

[39] Wu L., Tan Y., Liu Y. (2017). Factor structure and psychometric evaluation of the Connor-Davidson resilience scale in a new employee population of China. BMC Psychiatry.

[40] Ko C.-H., Yen J.-Y., Chen S.-H., Wang P.-W., Chen C.-S., Yen C.-F. (2014). Evaluation of the diagnostic criteria of Internet gaming disorder in the DSM-5 among young adults in Taiwan. J. Psychiar. Res..

[41] Connor K.M., Davidson J.R. (2003). Development of a new resilience scale: The Connor-Davidson resilience scale (CD-RISC). Depress. Anxiety.

[42] Wang L., Shi Z., Zhang Y., Zhang Z. (2010). Psychometric properties of the 10-item Connor–Davidson Resilience Scale in Chinese earthquake victims. Psychiatry Clin. Neurosci..

[43] McKenna KY A., West K.J. (2007). Give me that online-time religion: The role of the internet in spiritual life. Comput. Hum. Behav..

[44] Crumbaugh J.C., Maholick L.T. (1964). An Experimental Study in Existentialism: The Psychometric Approach to Frankl’s Concept of Noogenic Neurosis. J. Clin. Psychol..

[45] IBM Corp (2017). IBM SPSS Statistics for Windows 25.0.

[46] Johansson A., Grant J.E., Kim S.W., Odlaug B.L., Gotestam K.G. (2009). Risk factors for problematic gambling: A critical literature review. J. Gambl. Stud..

[47] Efron B., Tibshirani R. (1993). An Introduction to the Bootstrap.

[48] Aiken L.S., West S.G., Reno R.R. (1991). Multiple Regression: Testing and Interpreting Interactions.

[49] Cohen J., Cohen P., West S.G., Aiken L.S. (2003). Applied Multiple Regression/Correlation Analysis for the Behavioral Sciences.

[50] Huang G.-H. (2011). Responsible gambling policies and practices in Macao: A critical review. Asian J. Gambl. Issues Public Health.

[51] Institute for the Study of Commercial Gaming (ISCG) (2018). Responsible Gambling Awareness Survey 2017.

[52] Blaszczynski A., Nower L.A. (2002). pathways model of problem and pathological gambling. Addiction.

[53] King D., Delfabbro P., Griffiths M. (2010). Video Game Structural Characteristics: A New Psychological Taxonomy. Int. J. Ment. Health Ad..

[54] Shaffer H.J., Hall M.N. (2002). The natural history of gambling and drinking problems among casino employees. J. Soc. Psychol..

[55] Steger M.F., Mann J.R., Michels P., Cooper T.C. (2009). Meaning in life, anxiety, depression, and general health among smoking cessation patients. J. Psychosom. Res..

